# Erythropoietin Signaling Increases Choroidal Macrophages and Cytokine Expression, and Exacerbates Choroidal Neovascularization

**DOI:** 10.1038/s41598-018-20520-z

**Published:** 2018-02-01

**Authors:** Colin A. Bretz, Vladimir Divoky, Josef Prchal, Eric Kunz, Aaron B. Simmons, Haibo Wang, Mary Elizabeth Hartnett

**Affiliations:** 1 John A. Moran Eye Center, University of Utah, Salt Lake City, UT USA; 2 Department of Biology, Palacky University, Olomouc, Czech Republic; 3 Department of Hematology, University of Utah, Salt Lake City, UT USA

## Abstract

Erythropoietin (EPO) is recognized for neuroprotective and angiogenic effects and has been associated with aging and neovascular age-related macular degeneration (AMD). We hypothesized that systemic EPO facilitates the development of choroidal neovascularization (CNV). Wild type mice expressed murine EPOR (mWtEPOR) in RPE/choroids at baseline and had significantly increased serum EPO after laser treatment. To test the role of EPO signaling, we used human EPOR knock-in mice with the mWtEPOR gene replaced by either the human EPOR gene (hWtEPOR) or a mutated human EPOR gene (hMtEPOR) in a laser-induced choroidal neovascularization (LCNV) model. Loss-of-function hWtEPOR mice have reduced downstream activation, whereas gain-of-function hMtEPOR mice have increased EPOR signaling. Compared to littermate controls (mWtEPOR), hMtEPOR with increased EPOR signaling developed larger CNV lesions. At baseline, hMtEPOR mice had increased numbers of macrophages, greater expression of macrophage markers F4/80 and CD206, and following laser injury, had greater expression of cytokines CCL2, CXCL10, CCL22, IL-6, and IL-10 than mWtEPOR controls. These data support a hypothesis that injury from age- and AMD-related changes in the RPE/choroid leads to choroidal neovascularization through EPOR-mediated cytokine production.

## Introduction

Erythropoietin (EPO) is increasingly recognized for neuroprotective and angiogenic effects in addition to its well established role in hematopoiesis^1^. Clinical trials have tested or are testing forms of EPO as a neuroprotective factor in several conditions, including autoimmune optic neuritis, retinopathy of prematurity, and traumatic optic neuropathy^1^. In addition, EPO has been proposed to be protective in diabetic retinopathy, macular edema and even age-related macular degeneration (AMD)^1^, in which a hypothesis was posed that EPO, through its neuroprotective and anti-oxidative effects, may prevent progression of atrophic AMD^2^. In hematopoiesis, EPO binds to its receptor and triggers the JAK/STAT5 signaling cascade. Evidence suggests that EPO receptor (EPOR) can interact with other receptors to mediate non-hematopoietic effects involving angiogenesis or tissue protection. Interactions between EPOR and the beta-common receptor enable neuro- or tissue protective effects, whereas interactions between EPOR and vascular endothelial growth factor receptor 2 (VEGFR2) are believed to enable angiogenesis^3^. EPOR has been reported in several cell types within the eye including the RPE, photoreceptors, amacrine cells, bipolar cells, horizontal cells or ganglion cells^1,4,5^, thereby suggesting that direct ligand/receptor interactions are possible to exert neuroprotective or other effects in these cells. In addition to its direct effect on specific cells, EPO has been found to mediate indirect effects by binding receptors on other cell types^6^. For example, EPO exerts anti-neoplastic and immunomodulatory functions by mediating effects on lymphocytes^6^, which lack the EPOR, and does this by binding and activating EPOR on dendritic cells or macrophages^6^.

A previous study reported that greater numbers of circulating bone marrow-derived stem cells correlated with increased serum EPO levels in patients with neovascular AMD and compared to corresponding measurements in patients with inactive AMD. The investigators suggested that bone-marrow derived cells might be recruited through a signal released from active CNV and be involved in the pathogenesis of neovascular AMD^7^. Other investigators have found the importance of immune cells, such as tissue macrophages, in neovascular AMD^8–10^. We wished to investigate the role of EPO in choroidal neovascularization (CNV), a feature of neovascular AMD, and proposed the hypothesis that EPOR signaling affects choroidal macrophages causing them to facilitate the development of CNV through increased cytokine expression. Because controversy exists regarding the specificity of antibodies to EPOR^11,12^, we used unique humanized knock-in mice that either express the human EPOR gene (hWtEPOR) or a mutant truncated human EPOR gene (hMtEPOR) known to cause human polycythemia in place of the murine EPOR gene (mWtEPOR). Neither humanized mutant affects murine EPO/EPOR binding^13^, but rather causes reduced or enhanced signaling through reduced stability of a transmembrane protein necessary for EPOR dimerization and downstream signaling activation, in the case of the hWtEPOR^14^, or by lack of a feedback mechanism to reduce EPOR signaling, as in the hMtEPOR^13^. Knowledge from this study lends insight into the pathophysiology of neovascular AMD and may also be important when assessing safety in human clinical trials testing exogenous EPO for other conditions.

## Results

### Effect of Experimental CNV on Local and Systemic EPO Levels

**Figure 1:**
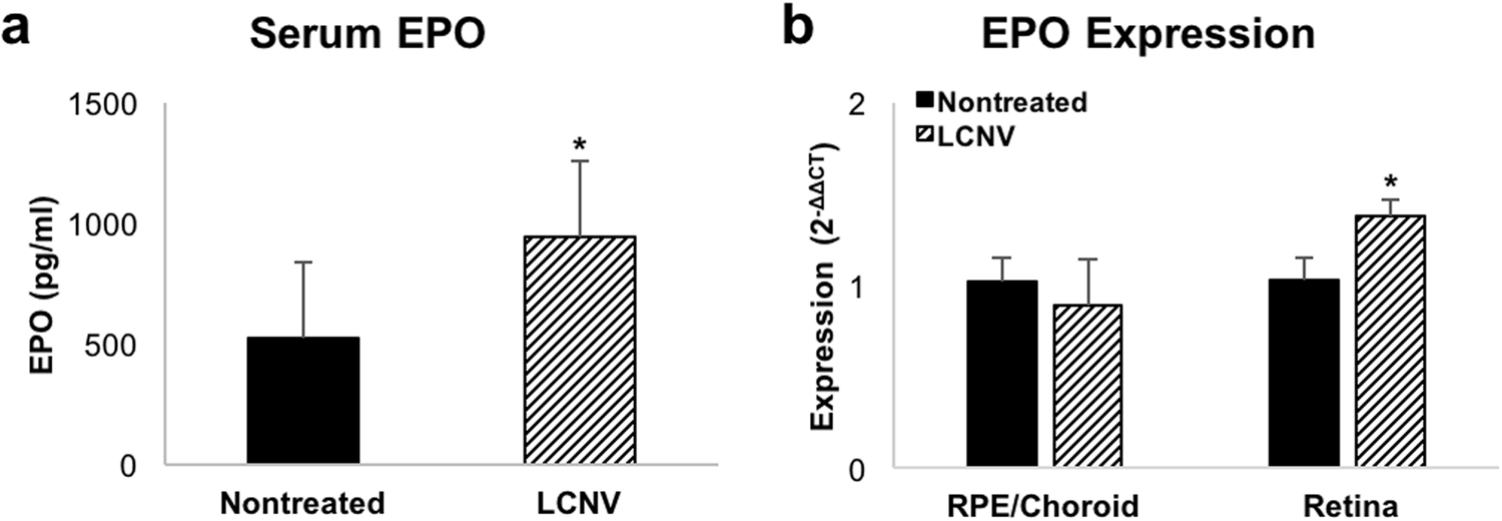
Effect of CNV treatment on local and systemic EPO levels. (**a**) Serum EPO levels in non-treated mWtEPOR mice and seven days following laser treatment (n = 11 and 9). (**b**) Expression levels of EPO mRNA in the RPE/choroids and retinas of non-treated and laser-treated mWtEPOR mice (n = 4–6 mice for each treatment group). Results are means ± SEM. *p < 0.05.

Humans with active neovascular AMD were reported to have increased numbers of circulating bone marrow cells, which positively correlated with serum EPO levels compared to patients with stable AMD^7^; therefore, we determined if serum EPO was elevated in C57Bl/6 J mice treated with laser to induce CNV as a model to study induced systemic EPO and CNV. Seven days after laser-induced injury, there was robust CNV. Compared to untreated age-matched control mice, serum EPO was increased in laser-treated mice (p = 0.015; Fig. 1a). We also measured EPO mRNA expression in both the RPE/choroid and retina of laser-treated and control mice. Although there was no difference in the choroidal expression levels, we did find an increase in retinal EPO levels seven days after laser-induced injury (p = 0.029; Fig. 1b). Our findings show that increased EPO temporally occurred in association with the development of experimentally-induced CNV supporting the use of the laser-induced CNV model to gain insight into the role of EPO in CNV.

### Effect of EPOR Signaling on Laser-Induced Choroidal Neovascularization

**Figure 2:**
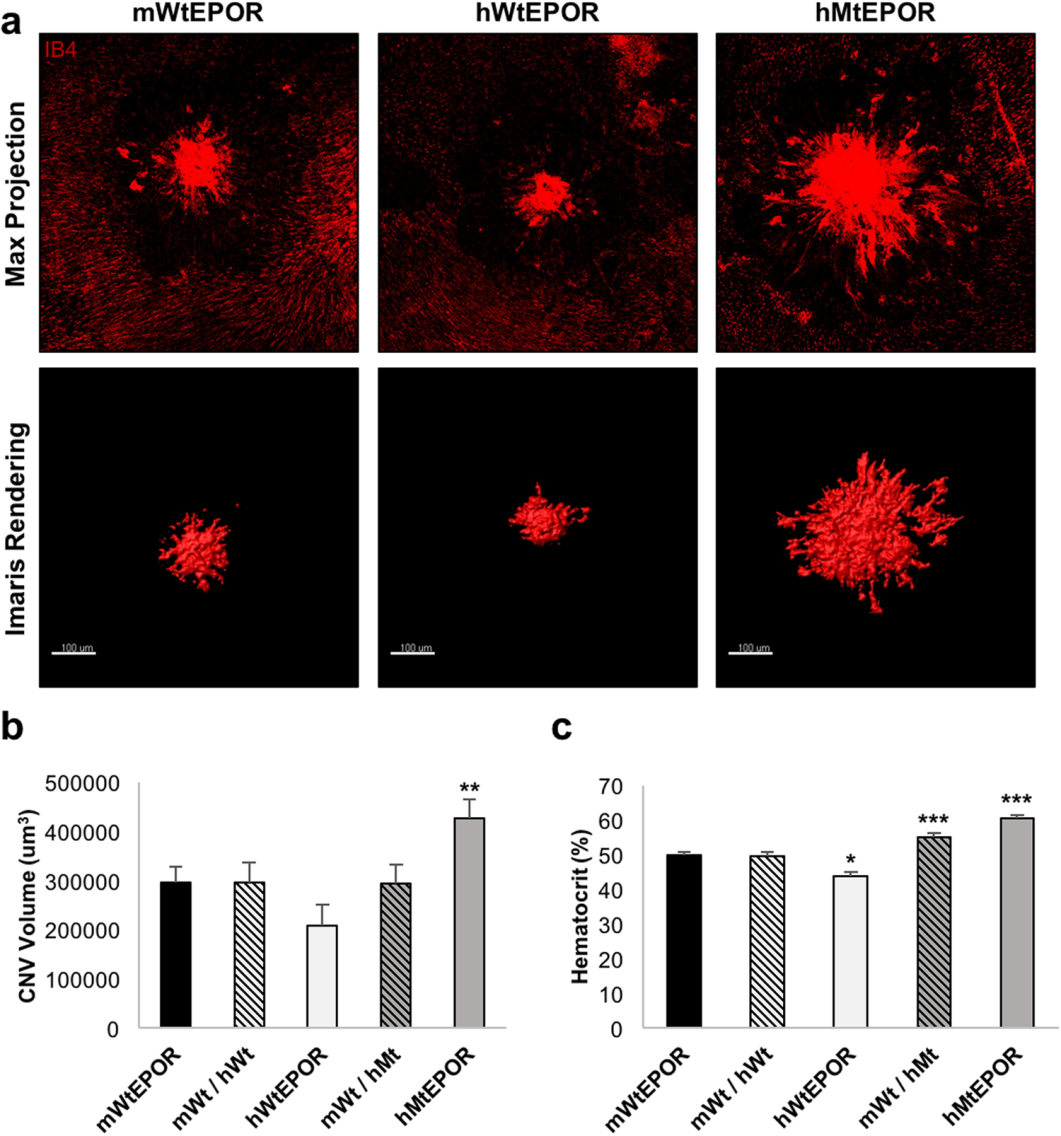
Effect of altered EPOR signaling on laser-induced choroidal neovascularization. (**a**) Representative images of individual lesions from mWtEPOR, hWtEPOR, and hMtEPOR mice seven days after laser treatment. Flatmounts were stained with IB4 and Z-stacks were captured at 20× and then rendered using the surfaces surpass feature in IMARIS to calculate volume. Both confocal maximum projections and the IMARIS 3-D surface rendering are provided for comparison. (**b**) Comparison of CNV volumes in mWtEPOR mice and human EPOR knock-in mice seven days after laser treatment (n > 50 spots for each treatment group). (**c**) Comparison of hematocrit levels in mWtEPOR mice and human EPOR knock-in mice (n ≥ 20 mice for each treatment group). Results are means ± SEM. *p < 0.05, **p < 0.01, ***p < 0.001 relative to mWtEPOR.

EPO signals through EPOR to mediate biologic effects, but there are difficulties with specificity of antibodies to measure EPOR and its activation^11,12^. To assess if systemic EPO could bind EPOR in cells within the eye, we measured mRNA of EPOR in wild-type C57Bl/6 J mice and found EPOR was expressed in both RPE/choroid and retina (data not shown). To determine if the association of increased serum EPO and CNV was related to EPOR signaling in the choroid, we used a humanized mouse model for EPOR signaling and compared these genetically modified mice to respective littermate control mice (mWtEPOR). The human EPOR knock-in mice (hWtEPOR) have the murine EPOR gene replaced by the human EPOR gene. This change leads to partial loss of gene function and hypoactive EPOR signaling compared to mWtEPOR mice^15^. The human mutant EPOR knock-in mice (hMtEPOR) have the murine EPOR gene replaced by a truncated form of the human EPOR gene that leads to EPOR gain-of-function and overactive EPOR signaling compared to mWtEPOR^13,16^. Seven days after laser, RPE/choroidal flatmounts from hypomorphic hWtEPOR mice had CNV volumes that did not differ significantly from the mWtEPOR controls (209114 ± 42759 vs. 295508 ± 32866 um^3^, p = 0.109; Fig. 2b). In contrast, gain-of-function EPOR mice, hMtEPOR, developed significantly increased CNV volumes compared to mWtEPOR control animals (427888 ± 37041 vs 295508 ± 32866, p = 0.008). These findings provide evidence that increased EPOR signaling is sufficient to increase CNV volume seven days after laser-induced injury.

### Hematocrits Do Not Align with Laser-induced Choroidal Neovascularization

EPO increases red blood cell production resulting in increased red cell mass that can be estimated by increased hematocrit. High hematocrit is found in human polycythemia and can be associated with reduced blood flow and reduced oxygenation to tissue beds. To investigate whether increased hematocrits in hMtEPOR were associated with differences in CNV volumes compared to mWtEPOR mice, we measured hematocrits at the time of tissue harvest in all age-matched animals. Similar to previous studies using these knock-in mice^13^, hWtEPOR mice had significantly lower hematocrits than mWtEPOR mice, and hMtEPOR mice had elevated hematocrits compared to the mWtEPOR (p < 0.001 and p < 0.001; Fig. 2c). When the results were analyzed by sex (Table 1), female hMtEPOR had significantly lower hematocrits than male hMtEPOR mice (p < 0.001) but developed nearly identical CNV volumes. We also measured hematocrits of heterozygous animals for each of the humanized mutant mouse models. There was no difference in CNV volumes between either heterozygous genotype compared to respective wild type control littermates (Fig. 2b), even though hematocrits were significantly higher in the hMtEPOR heterozygous mice compared to homozygous mWtEPOR control mice (p < 0.001). Both of these findings suggest that increased hematocrit was not the principal reason for differences in CNV volumes.

**Table 1:** Effect of Genotype and Sex on Lesion Volume, Hematocrit, and Choroidal Macrophages Numbers in hEPOR Knock-in Mice.

Genotype	Murine Model Phenotype	Average Lesion Volume (um^3^)			Hematocrit (%)			Choroidal MacrophagesPer Field
		Average	Male	Female	Average	Male	Female	
mWtEPOR	Normal EPOR signaling	295508 ± 32866	297985 ± 51844	293074 ± 41786	50.02 ± 0.65	50.31 ± 0.88	49.73 ± 0.88	304 ± 12
mWT/hWT	—	296479 ± 40795	259099 ± 47478	389931 ± 74636	49.51 ± 1.15	50.36 ± 1.32	47.57 ± 1.99	—
hWtEPOR	Reduced EPOR signaling	209114 ± 42759	187633 ± 55936	234682 ± 63520	43.83^***^ ± 1.23	45.36^*^ ± 1.76	42.57^***^ ± 1.99	328 ± 14
mWT/hMT	—	294178 ± 38493	311881 ± 49956	271356 ± 57407	54.94^***^ ± 1.21	56.75^**^ ± 2.16	54.22^**^ ± 1.36	—
hMtEPOR	Increased EPOR signaling	427888^**^ ± 37041	438739^*^ ± 55936	421238^*^ ± 47888	60.44^***^ ± 0.99	63.56^***^ ± 1.28	56.64^***#^ ± 1.41	373^***^ ± 30

*Significantly different from mWtEPOR control; *p < 0.05, **p < 0.01, ***p < 0.001. ^#^Significantly different from males of same genotype; ^#^p < 0.001.

### Choroidal Macrophages are Increased by Augmented EPOR Signaling

**Figure 3:**
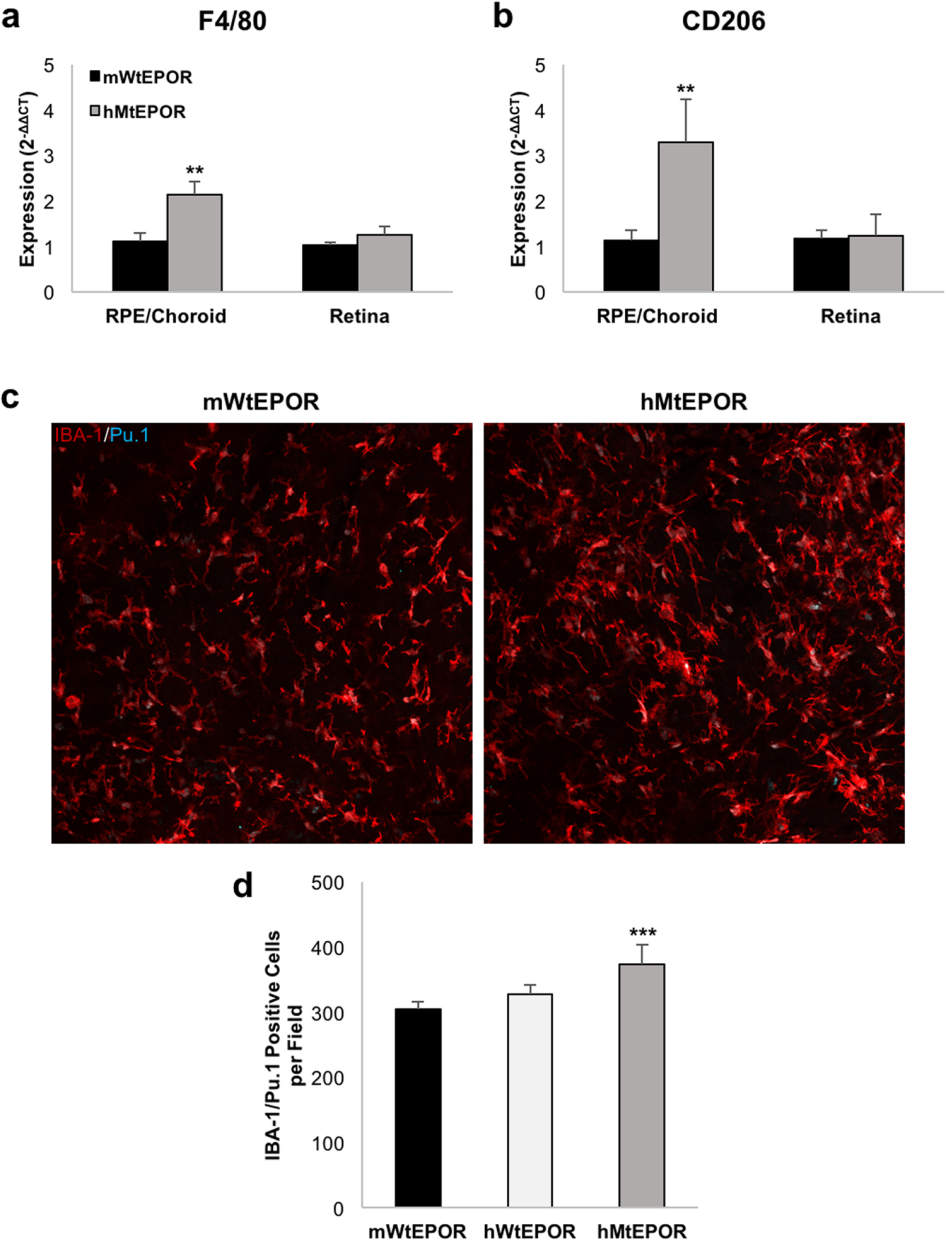
Effect of increased EPOR signaling on macrophage markers in ocular tissue. (**a**) and (**b**) mRNA expression levels of macrophage markers (**a**) F4/80 and (**b**) CD206 in the RPE/choroids and retina of non-treated mWtEPOR and hMtEPOR mice (n ≥ 6 animals per treatment group). (**c**) Representative images of IBA-1/Pu.1 positive cells in choroidal tissue from mWtEPOR and hMtEPOR mice. (**d**) Comparison of the number of IBA-1/Pu.1 cells per image (n = 8–16 images per genotype). Results are means ± SEM. **p < 0.01, ***p < 0.001 relative to mWtEPOR non-treated control.

Macrophages are important in human and experimental CNV^6,10,17–19^, and are known to be activated by EPO in other tissue beds^6,20^. Therefore, we evaluated whether macrophages were more prevalent in the retinas and choroids of hMtEPOR mice, which had larger CNV lesions, than in mWtEPOR control littermates. As one method to quantify macrophages in each of the two genotypes, we measured the expression of macrophage markers F4/80 and CD206 in both retinal and RPE/choroidal tissue from non-lasered animals by real-time PCR (RT-PCR). hMtEPOR RPE/choroids had increased gene expression of both F4/80 and CD206 compared to mWtEPOR controls (p = 0.009 and p = 0.006; Fig. 3a,b), but there was no difference in expression levels of these macrophage markers in the retinas of the two genotypes. As another investigation, we stained RPE/choroidal flatmounts from untreated eyes of mWtEPOR, hWtEPOR, and hMtEPOR mice with antibodies against macrophage marker IBA-1 and the macrophage nuclear marker Pu.1, and quantified the number of IBA-1/Pu.1 positive macrophages (Fig. 3c,d). Consistent with our mRNA expression analysis, flatmount staining revealed that hMtEPOR animals had increased numbers of macrophages in the choroid compared to mWtEPOR (p = 0.006), whereas hWtEPOR choroids had macrophage numbers that did not significantly differ from mWtEPOR.

### The Effect of Increased EPOR Signaling on Macrophages in CNV

**Figure 4:**
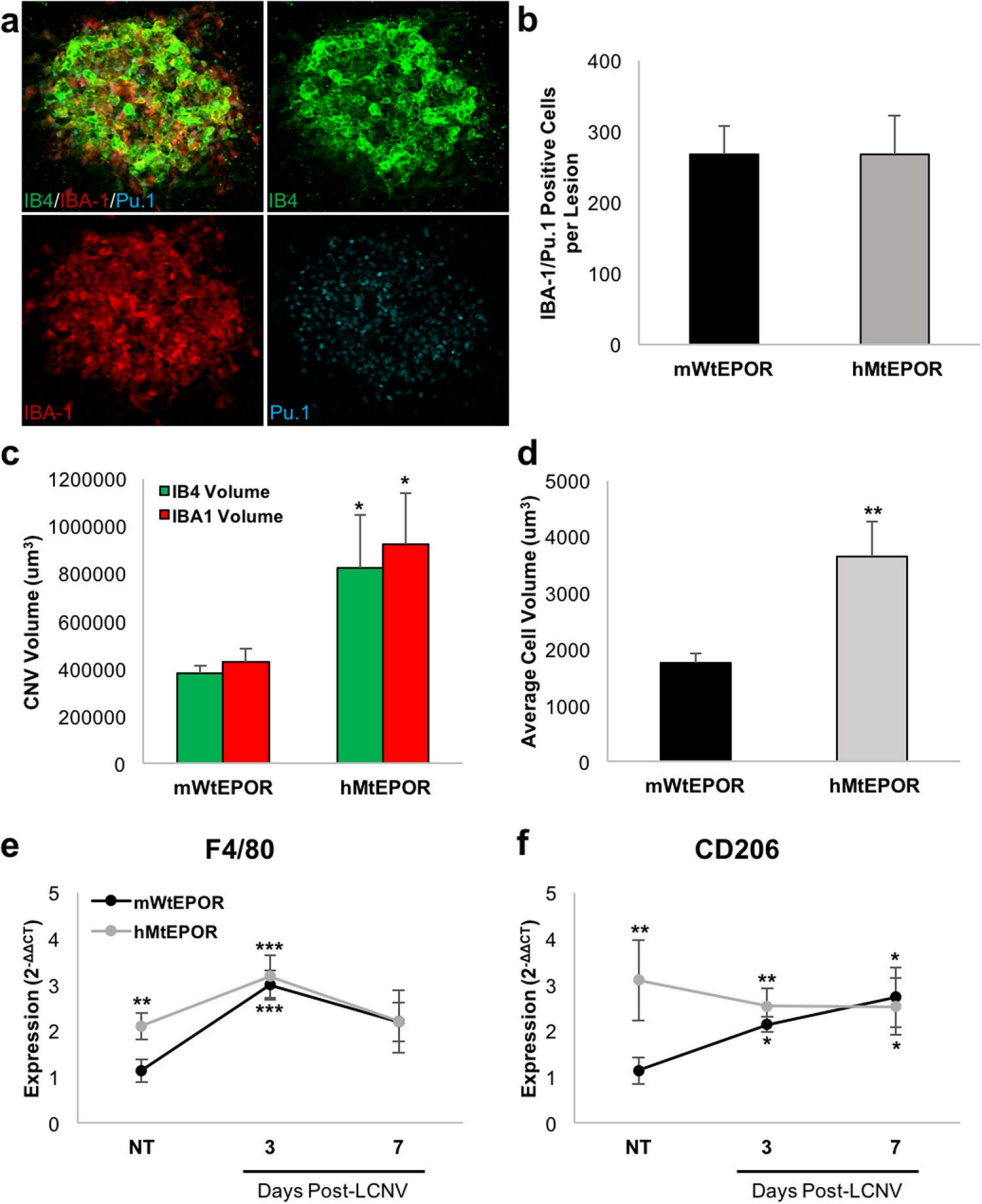
Effect of increased EPOR signaling on LCNV lesions three days post laser treatment. (**a**) Representative images of CNV staining at three days post-laser. (**b**) Comparison of the number of IBA1/Pu.1 positive cells per CNV spot (n ≥ 12 spots per group). (**c**) Comparison of CNV volumes in mWtEPOR and hMtEPOR three days after laser treatment (n ≥ 12 spots per group). (**d**) Estimate of average macrophage cell volume within a CNV spot (n ≥ 12 spots per group) (**e**) and (**f**) mRNA expression levels of macrophage markers (**e**) F4/80 and (**f**) CD206 at three and seven days post-laser treatment (n = 5–8 animals per treatment group). Results are means ± SEM. *p < 0.05, **p < 0.01, ***p < 0.001 relative to mWtEPOR non-treated control.

Since the expression of macrophage markers and the number of macrophages were increased in the RPE/choroids of untreated hMtEPOR mice, we asked whether increased CNV volumes at seven days post-laser in hMtEPOR RPE/choroids were associated with early recruitment to and later activation of macrophages at the sites of injury. Others have reported that macrophage infiltration peaks between one and three days after laser-induced injury^10,21^, so we counted the number of macrophages that were co-labeled with IBA-1 and Pu.1 in lesions of hMtEPOR and mWtEPOR mice three days after laser and found a significant number of IBA1/Pu.1 positive cells had infiltrated the CNV (Fig. 4a). However, there was no significant difference in the number of IBA1/Pu.1 positive cells between laser-induced CNV of hMtEPOR and mWtEPOR (Fig. 4b), despite increased CNV volumes in the hMtEPOR animals (p = 0.04; Fig. 4c). Of note, volume measurements were similar for individual lesions using either IB4 (As in Fig. 2) or IBA-1 staining. Using the IBA-1 volume measurements and Pu.1 nuclei counts we were able to estimate the average macrophage cell volume within the lesion, and found that macrophages in laser-treated hMtEPOR mice had significantly larger cell volumes compared to mWtEPOR (p = 0.009; Fig. 4d). These data suggest that at three days post-laser, CNV was composed largely of macrophages and inflammatory cells, consistent with previous studies^22^.

As an additional measurement of macrophage infiltration, we determined mRNA expression levels of macrophage markers F4/80 and CD206 (Fig. 4e,f). Baseline levels of F4/80 and CD206 were lower in mWtEPOR than hMtEPOR mice but rose to expression levels similar to those of hMtEPOR mice three and seven days after laser. These findings were consistent with our observations in stained tissue, that hMtEPOR mice have increased numbers of choroidal macrophages in non-injured tissue compared to mWtEPOR, but do not experience increased infiltration of macrophages compared to mWtEPOR three days following laser treatment.

### The Effect of Increased EPOR Signaling on Inflammatory Cytokines in RPE/choroid

Given the observation that hMtEPOR lesion volumes were larger than mWtEPOR lesions at three days post-laser, but there was no difference in cell number or expression of macrophages markers, we asked if macrophages might be activated to produce more cytokines and thereby contribute to greater lesion volume in the hMtEPOR mice. Previous studies report that EPO activates macrophages^6^, can prime peripheral blood mononuclear cells to produce inflammatory cytokines^20^ and exert an immunoregulatory effect on macrophages^23^. The literature on AMD also reports increased serum and vitreous biomarkers of activated macrophages in patients with AMD^24–28^. Therefore, to evaluate whether increased EPOR signaling in the hMtEPOR mice led to increased expression of inflammatory cytokines associated with AMD and known to drive LCNV development, we measured the mRNA expression at three and seven days post-laser treatment of several cytokines in RPE/choroids, particularly those cytokines produced by macrophages and previously reported in association with neovascular AMD^10^.

**Figure 5:**
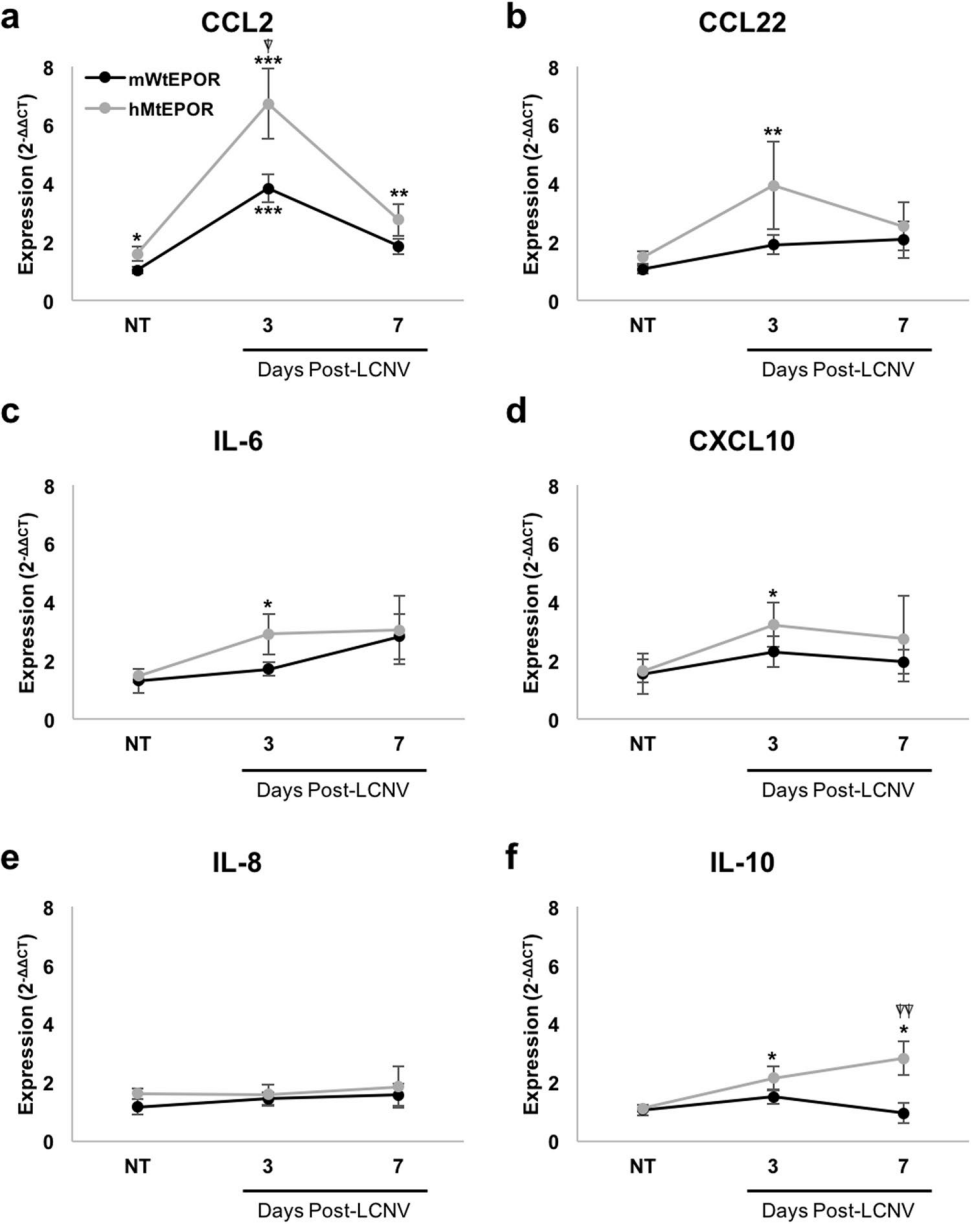
Effect of increased EPOR signaling on chemokine expression in RPE/Choroid. (**a–f**) mRNA expression levels of inflammatory chemokines (**a**) CCL2 (**b**) CCL22 (**c**) IL-6 (**d**) CXCL10 (**e**) IL-8 (**f**) IL-10 in RPE/Choroids of non-treated and laser-treated mWtEPOR and hMtEPOR mice (n = 5–8 animals per treatment group). Results are means ± SEM. *p < 0.05, **p < 0.01, ***p < 0.001 relative to mWtEPOR non-treated control, ^⍒^p < 0.05. ^⍒⍒^p < 0.01 relative to mWtEPOR treated control.

Expression of the monocyte chemoattractant, CCL2, was increased in both mWtEPOR and hMtEPOR tissue three days after laser compared to non-treated control mWtEPOR (p < 0.001 mWtEPOR; p < 0.001, hMtEPOR; Fig. 5a), and CCL2 was significantly increased at day three in laser-treated hMtEPOR compared to laser-treated mWtEPOR tissue (p = 0.034). Compared to non-treated mWtEPOR controls, CCL2 expression was also significantly increased in non-treated hMtEPOR RPE/choroids (p = 0.048) and at seven days after laser (p = 0.003). Chemokines CCL22 (p = 0.008; Fig. 5b), IL-6 (p = 0.029; Fig. 5c), and CXCL10 (p = 0.038; Fig. 5d) were all significantly increased in hMtEPOR tissue, but not in mWtEPOR, three days after laser compared to non-treated mWtEPOR control. IL-10 was upregulated in hMtEPOR tissue at both three and seven days after laser treatment compared to mWtEPOR control (p = 0.034 three days and p = 0.032 seven days; Fig. 5f), and was also significantly increased relative to laser-treated mWtEPOR at day seven (p = 0.004). There was no difference in IL-8 expression in either genotype at any time point (Fig. 5e). Therefore, compared to mWtEPOR, augmented EPOR signaling in the hMtEPOR mice resulted in increased expression of several inflammatory cytokines following laser treatment that have been reported in association with macrophage activation and human neovascular AMD.

## Discussion

EPO has been proposed for its neuroprotective and anti-oxidative effects as a potential preventive treatment in various diseases, including in atrophic AMD^2^. However, there remain concerns about possible pathologic effects from other effects of EPO, including angiogenesis and its potential role in cancer progression^29,30^. In this study, we investigated the role of EPO/EPOR signaling in experimental CNV and explored the hypothesis that EPOR signaling would lead to the development of CNV. We found that increased EPOR signaling increased injury-induced CNV, independent of hematocrit, potentially through the recruitment of macrophages to the choroid where they released cytokines and facilitated the development of CNV.

AMD is complex and evidence is accumulating about the importance of inflammatory events that precede angiogenic factors and angiogenesis. Besides the role of macrophages, it is recognized that mast cells are also important mediators of inflammation and cytokine release in human AMD^9^. Our study supports the premise that the choroid is an immune site and if macrophages are recruited to it, the events surrounding the development of CNV can be enhanced.

Using the laser-induced injury model in C57Bl6/J mice, we initially found serum EPO was increased seven days following laser treatment in association with the formation of CNV. In addition, retinal EPO expression was elevated following laser and may be involved in triggering EPOR signaling in the choroid. Murine RPE/choroids from laser-treated eyes also had increased expression levels of two macrophage markers, F4/80 and CD206, compared to untreated mWtEPOR eyes. Previous investigators found serum EPO to be modestly increased in patients with active neovascular AMD compared to inactive AMD^7^. Even though serum EPO was not significantly different between active and stable AMD, the correlation between EPO and circulating bone-marrow derived CD34+ endothelial progenitor cells was significant in their study, whereas other factors (VEGF, SDF-1 alpha, Ang-1) were not correlated. In our study, we specifically looked at macrophages, which are produced from bone-marrow derived monocytes. Previous investigators also explored how EPO affected the immune status of patients with multiple myeloma and found that the immunomodulatory effects of EPO on lymphocytes, which lack the EPOR, were indirect and occurred by activating macrophages that express EPOR^6^. In addition, a number of investigators have shown that macrophages play an important role in the development of experimental CNV^31,32^, and along with other immune-related cells, are critical features in the development of human neovascular AMD^8–10,18,19,33,34^. We were interested in the possibility that macrophages, as immune cells, mediated the effects of EPO on CNV through EPOR signaling.

Previous studies have focused on the EPO hormone/ligand and not on the signaling effects through EPOR. Part of the reason may be that immunolocalizing the EPOR is difficult due to poor EPOR antibody specificity^11,12^. We determined that the EPOR was expressed in the retina, RPE and choroid in murine wild type C57Bl/6 J mice. Then, to explore the premise that EPO-triggered signaling affected macrophages in CNV, we used hypomorphic and gain-of function EPOR knock-in mice to evaluate the effects of altered EPOR signaling. Due to extensive homology and biological cross-reactivity between human and murine EPO and EPOR, both human EPOR genes are able to efficiently bind mouse EPO^35,36^. However, the hWtEPOR mice have reduced EPOR signaling due to decreased stability of a transmembrane domain^14^, whereas the hMtEPOR mice have overactive EPOR signaling due to a truncation in the cytosolic negative regulatory domain of the receptor^13^. These unique models allow us to test the effect of EPOR signaling on biologic outcomes independently of the EPO ligand. EPOR signaling can regulate EPO expression, so compared to mWtEPOR mice, the hMtEPOR mice with increased EPOR signaling have reduced EPO ligand and the hWtEPOR mice with reduced EPOR signaling have elevated EPO ligand^13^. To evaluate the role of EPOR signaling in LCNV, we treated control and knock-in mice with laser and found that hMtEPOR mice developed significantly larger CNV volumes than either hWtEPOR or control mice. One reason might have been related to increased hematocrit and reduced tissue oxygenation. EPO is a well-established regulator of hematopoiesis, and the EPOR knock-in mice have previously been shown to have altered hematocrit levels^16^. Consistent with previous studies, we found that homozygous hWtEPOR mice had reduced hematocrit levels compared to mWtEPOR mice, whereas hMtEPOR mice had significantly increased hematocrit levels. However, hMtEPOR heterozygote mice also had significantly higher hematocrit levels compared to mWtEPOR controls, but there was no difference in CNV volumes between the genotypes. Also, female hMtEPOR mice had significantly lower hematocrit levels than male hMtEPOR mice, but both males and females had similarly increased CNV volumes. Taken together, these findings suggest that differences in hematocrit did not account for differences seen in CNV volumes.

Given the importance of macrophages in the development of CNV and reports that EPO is involved in the activation of macrophages in other tissue beds, we evaluated whether increased EPOR signaling in the hMtEPOR mice had an effect on macrophage number or activation in the choroid. mRNA analysis showed that hMtEPOR mice had increased RPE/choroid expression of the macrophage markers F4/80 and CD206, as well as increased numbers of choroidal IBA-1/Pu.1 macrophages compared to mWtEPOR. CCL2 is a macrophage chemoattractant, and the increased expression at baseline in hMtEPOR tissue compared to mWtEPOR corresponds with the increased numbers of macrophages. However, there was no difference in the expression of F4/80 or CD206 in the retinal tissue of hMtEPOR and mWtEPOR mice. (There was also no difference in the number of choroidal macrophages found in hWtEPOR and mWtEPOR mice, which may explain why there was no significant difference in the lesion volume of hWtEPOR mice). Despite increased numbers of macrophages in the untreated hMtEPOR choroid, there was no difference in the number of macrophages found in the CNV lesions in hMtEPOR mice three days after laser. We interpret this to mean that hMtEPOR mice have a greater number of choroidal macrophages available to respond to tissue injury compared to mWtEPOR mice. Following laser, the hMtEPOR macrophages were then activated to release more cytokines compared to baseline, but laser did not lead to greater recruitment of macrophages to CNV lesions in the hMtEPOR mice at three days. There may be additional recruitment at later time points. Perhaps the larger volume of CNV resulted from a faster response from existing macrophages in the choroid of hMtEPOR mice compared to mWtEPOR. This observation aligned with the analyses of mRNA in which the expression levels of F4/80 and CD206 in mWtEPOR mice increased to the expression levels of hMtEPOR three and seven days after laser.

Although macrophage cell number was not different between hMtEPOR and mWtEPOR mice after laser, CNV volumes were increased in the hMtEPOR mice. We postulated that increased EPOR signaling was related to activation of macrophages to produce inflammatory cytokines that drive CNV formation. To address this hypothesis, we measured the expression of a number of cytokines that are important in experimental and clinical AMD-related CNV, namely CCL2^37^, CCL22^10^, CXCL10^10^, IL-6^10^ and IL-10^38,39^, and found that their expression levels were significantly increased in laser-treated hMtEPOR mice compared to non-treated controls. CCL2 and IL-10 were also both increased in hMtEPOR mice relative to laser-treated mWtEPOR. Both CCL2 and Il-10 are known to be critical macrophage signaling molecules in the development of CNV; CCL2 and IL-10 were increased in choroidal tissue following laser-induced CNV^10^, and both CCL2 and IL-10 knockout mice had reduced laser-induced CNV compared to controls^31,40^. Previous studies also identified roles for EPO in their production. EPO-treated EPOR-positive peripheral blood mononuclear cells secreted more IL-10 in response to injury compared to non-EPO treated cells^20^. Another group reported that exogenous EPO upregulated CCL2 and activated CCL2 signaling in cardiac progenitor cells^41^. These studies suggest that EPOR signaling can trigger inflammatory and other cells to produce cytokines in response to tissue injury. Besides the effect of EPOR signaling on inflammatory cells, there is also evidence of the importance of EPOR signaling and of inflammation in causing angiogenesis within the eye^42–46^.

In addition to our hypothesis testing EPOR signaling’s role in choroidal macrophages and CNV, many have reported on the role of chemokine expression on macrophages in CNV. This is beyond the scope of this report but is important to recognize nonetheless as the pathophysiology of neovascular AMD is complex. Evidence supports the role of macrophages in the pathogenesis of neovascular AMD. Previous studies reported that depletion of choroidal macrophages resulted in smaller CNV lesions, as does knockout of macrophage derived chemokines such as CCL2^40^. However, other studies report that macrophages can inhibit CNV^31^, and it partly depends on the phenotype of the macrophage. Based on reports in the literature, this seems to depend on the polarization of macrophages, which is based on expression profile of chemokines and surface markers (eg., classical macrophage activation (M1) and alternatively activated macrophages (M2)). Immediately following laser-induced injury, macrophages tend toward an M1 phenotype^10^, which inhibits CNV^31^. In the presence of IL-10 and other cytokines, macrophages can become alternatively activated and take on a pro-angiogenic M2 phenotype, characterized by CCL2 and CCL22 expression. M2 macrophages have previously been associated with a pro-angiogenic phenotype^47^. We found higher expression of several M2 markers (CCL2 and CCL22) in hMtEPOR mice, and this expression profile aligns with our observation that hMtEPOR mice have significantly larger CNV than mWtEPOR mice.

Increases in CCL2 and IL-10 are also both associated with aging in the RPE/choroid. Pro-inflammatory CCL2-CCR2 signaling in the choroid increased with age in mice^31,37^, and other investigators reported that older mice had increased levels of IL-10 both systemically and in the RPE/choroid^38,39^. Furthermore, compared to younger similarly-injured mice, older mice developed larger CNV volumes in response to laser-induced injury in association with increased IL-10 secretion from macrophages^39^. Serum EPO has been reported as increased in older age^48^ and in association with components of metabolic syndrome, which has been associated with late AMD^49^. Although speculative, EPO may be released with increasing age as a protective agent or to compensate for increased subclinical red blood cell loss or turnover and may then act as a factor that primes the choroid to facilitate the development of CNV. Our study also adds concern about the risk associated with systemically delivered EPO as a performance enhancing drug or in clinical trials for diseases associated with macrophage mediated angiogenesis, such as neovascular AMD.

In summary, our study supports a hypothesis that EPOR signaling leads to recruitment and activation of macrophages to tissue beds, such as the choroid, which is prone to inflammation. This aligns with previous work suggesting that a signal from human CNV caused bone-marrow derived cells to be mobilized. We propose that injury from aging and other stressors related to AMD, and possibly with genetic predisposition, leads to release of signal(s) that either directly or indirectly trigger EPO release to activate macrophages and cause them to move to the choroid, priming it for CNV formation. More experimental and clinical studies are needed to test this hypothesis, but as clinical trials are being performed to test the role of EPO as a neuroprotective agent, attention to the effects on the choroid and AMD become increasingly important.

## Methods

### Animals

Animal protocols were approved by IACUC and the Institutional Biosafety Committee of the University of Utah. All animal procedures were conducted in accordance with University of Utah (Guide for the Care and Use of Laboratory Animals) and the Association for Research in Vision and Ophthalmology Statement for the Use of Animals in Ophthalmic and Vision Research. All experiments included both male and female mice between six and seven weeks of age. The humanized mouse model for EPOR signaling consisted of two knock-in mice with either the hWtEPOR or hMtEPOR gene knocked into the endogenous mouse *EpoR* locus were developed by gene targeting and then backcrossed to the C57BL/6 background^13^. hWtEPOR mice have hypoactive EPOR signaling due to a loss of gene function, where-as hMtEPOR mice have increased EPOR signaling due to a gain-of-function mutation. All mice are on a C57Bl/6 J background.

### Quantification of Serum Cytokines Using ELISA

Serum was collected and spun down for 25 min at 2000 × g. The supernatant was collected and assayed as recommended using a mEPO ELISA (R&D, Minneapolis, MN). Each sample was run in duplicate and the average concentration was used for analysis. n ≥ 9 mice per group.

### Quantitative RT-PCR

Eyes from mWtEPOR and hMtEPOR mice were harvested prior to laser treatment and at three and seven days post-laser treatment. Eyes were washed in Phosphate Buffered Saline (PBS; Genesee Scientific, Sand Diego, CA) then immediately dissected. All muscle and excess tissue was removed from the posterior portion of the eye, in addition to the cornea, lens and vitreous. Lastly, the retina and RPE/choroid were separated and flash frozen separately. For RT-PCR, tissue was placed in buffer RLT and sonicated prior to mRNA isolation using a Qiagen RNEASY kit (Qiagen, Valencia, CA). cDNA was reverse transcribed from mRNA samples and evaluated using Taqman Gene Expression Assays (ThermoFisher Scientific, Waltham, MA). ΔCT values for target genes were calculated using both *β-actin* and *Tata-Box Binding Protein* as house-keeping controls and the 2^−ΔΔCT^ was calculated relative to the non-treated mWtEPOR group. n = 5–8 mice per group.

### Non-Treated Choroidal Macrophage Counts

Eyes from six-week-old mWtEPOR, hWtEPOR, and hMtEPOR mice were harvested and fixed in 4% paraformaldehyde for one hour. After removal of cornea, lens and vitreous, posterior eyecups of the RPE/choroid/sclera were dissected. Eyecups were incubated in blocking buffer (PBS with 0.01% Triton, 10% bovine serum albumin, and 10% normal goat serum), for one hour at room temperature, then stained with a primary antibody against IBA-1 (Rabbit anti-mouse; Abcam, Cambridge, UK) for 48hrs at 4 °C, prior to incubation with secondary antibody (Goat anti-rabbit TRITC; Invitrogen) and a conjugated Pu.1 antibody (Rat anti-mouse 657-conjugated; BioLegend, San Diego, CA) for an additional 48hrs at 4 °C. Stained RPE/choroid/sclera flatmounts were mounted scleral side up in Fluoromount-G mounting medium (SouthernBiotech, Birmingham, AL), and z-stacks were captured using a 20× objective and confocal microscope (FV1000, Olympus, Japan). Z-stacks spanned the entire depth of the flatmount, sampling at 1.26 µm between serial sections and were taken of four non-overlapping areas directly adjacent to, but not containing the optic nerve head. The number of IBA-1/Pu.1 positive cells per Z-stack were counted by hand using ImageJ software. n = 8–16 z-stacks per genotype.

### Laser-Induced Choroidal Neovascularization

Six-week-old hWtEPOR, hMtEPOR and litter mate control mWtEPOR mice were anesthetized with intraperitoneal injections of 100 mg/kg ketamine (Bioniche Teoranta, Ireland) and 10 mg/kg xylazine (Akorn, IL) following mydriasis with 0.5% tropicamide ophthalmic solution (Bausch & Lomb, NY). Mice were raised onto a platform in front of the Phoenix Micron IV Imaging System (Phoenix research Labs, Pleasanton, CA), and a coupling agent, GenTeal (Novartis, NJ), was applied to the cornea. Laser photocoagulation was performed using the Micron laser module (450 mW intensity, 100 ms duration; Phoenix Research Labs, Pleasanton, CA). Three laser spots per eye were applied approximately 2 disc diameters from the optic nerve, avoiding major vessels. Disruption of Bruch’s membrane was confirmed by the appearance of a cavitation bubble. Eyes were collected three and seven days post-laser and used for either flatmounts or RT-PCR.

### Analysis of Lesion Volume in RPE/Choroidal Flatmounts at Day Seven

At harvest, eyes were fixed, dissected, and blocked as described above, prior to being stained with AlexaFluor 568-conjugated IB4 (GS-IB4; Invitrogen) overnight at 4 °C. Eyecups were then mounted sclera side down and confocal Z-stacks of individual CNV spots were taken using the 20× objective and a 3 µm step size. Each CNV spot was measured both by hand and using the surfaces feature in IMARIS (Bitplane, Zurich, Switzerland). Hand counts were done using ImageJ software to measure the area of the lesion in each optical section. The total area of each lesion was then multiplied by the distance between images (3 µm) in order to calculate the volume of a given lesion. Z-stacks were also imported into IMARIS software and volumes were calculated by generating a 3-D rendering of the CNV spot using the surfaces feature. Statistical analysis was run on both the hand counted and IMARIS data sets and there was no discrepancy found between the outcomes. Eyes in which a hemorrhage during laser was observed, or in which bridging CNV developed, were excluded from all analysis. Each CNV spot was considered an independent data point, and n ≥ 50 CNV spots for each genotype, comprising at least n ≥ 20 for each sex.

### Hematocrit

Serum was collected during tissue harvest using heparin-treated micro-hematocrit tubes (VWR, Radnor, PA). The tubes were spun for 5 minutes in a hematocrit microcentrifuge, and read using a capillary micro-hematocrit reader. n was >20 animals for each treatment group.

### Analysis of Lesion Volume and Macrophage Numbers in RPE/Choroidal Flatmounts at Day Three

For CNV macrophage counts, eyes were fixed and dissected as described above. Dissected eyecups were incubated with IBA-1 primary antibody for 72 hrs at 4 °C (Rabbit anti-mouse), prior to incubation with secondary antibody (Goat anti-rabbit TRITC), IB4 (488-conjugated), and Pu.1 (Rat anti-mouse 657-conjugated). Labeled eyecups were mounted sclera side down and captured via confocal using the 20× objective at 1.26 µm Z-stack sections. IBA-1/Pu.1 double positive cells were counted by hand, using ImageJ software, and CNV volumes were calculated using IMARIS. Average macrophage cell volume was calculated by dividing the IBA-1 volume of an individual lesion by the number of Pu.1 nuclei within that lesion. n ≥ 12 CNV spots for each genotype.

### Statistics

All statistical analysis were run using STATA14 software (StataCorp, College Station, TX). ELISA and RT-PCR data were analyzed using a linear regression model, and RT-PCR statistics were run using the non-transformed ∆CT value for each sample. CNV volumes and hematocrit were analyzed using a multivariable mixed effects linear regression model to account for variability between litters, individual animals, and lesions within a given eye. p < 0.05 was considered statistically significant.

### Data Availability

The data sets generated and analyzed during this study are available from the corresponding author on reasonable request.
